# Enzyme-Assisted Coextraction of Phenolics and Polysaccharides from *Padina gymnospora*

**DOI:** 10.3390/md22010042

**Published:** 2024-01-12

**Authors:** Hoang Chinh Nguyen, Kim Ngan Ngo, Hoai Khang Tran, Colin J. Barrow

**Affiliations:** 1 Centre for Sustainable Bioproducts, Deakin University, Geelong, VIC 3216, Australia; 2 Faculty of Applied Sciences, Ton Duc Thang University, Ho Chi Minh City 700000, Vietnam; ngokimngan.0711@gmail.com (K.N.N.); tranhoaikhang@tdtu.edu.vn (H.K.T.)

**Keywords:** enzyme, simultaneous extraction, *Padina gymnospora*, optimization, antioxidant

## Abstract

Brown seaweed is a promising source of polysaccharides and phenolics with industrial utility. This work reports the development of a green enzyme-assisted extraction method for simultaneously extracting polysaccharides and phenolics from the brown seaweed *Padina gymnospora*. Different enzymes (Cellulast, Pectinex, and Alcalase), individually and in combination, were investigated, with Alcalase alone showing the highest efficiency for the simultaneous extraction of polysaccharides and phenolics. Yields from Alcalase-assisted aqueous extraction were higher than those obtained using either water alone or conventional ethanol extraction. Alcalase-assisted extraction was subsequently optimized using a response surface methodology to maximize compound recovery. Maximal polysaccharide and phenolic recovery was obtained under the following extraction conditions: a water-to-sample ratio of 61.31 mL/g, enzyme loading of 0.32%, temperature of 60.5 °C, and extraction time of 1.95 h. The extract was then fractionated to obtain alginate-, fucoidan-, and phenolic-rich fractions. Fractions exhibited potent 2,2-diphenyl-1-picrylhydrazyl radical scavenging activity with IC_50_ values of 140.55 µg/mL, 126.21 µg/mL, and 48.17 µg/mL, respectively, which were higher than those obtained from conventional extraction methods. The current work shows that bioactive polysaccharides and phenolics can be obtained together in high yield through a single aqueous-only green and efficient Alcalase-assisted extraction.

## 1. Introduction

Cellular oxidative stress and autoxidation contribute to the ageing process in humans and are implicated in the development of various chronic disorders including cardiovascular disease, cancer and diabetes. Synthetic antioxidants are commonly used in food and health products, although rising concern about the side effects of synthetic antioxidants has restricted their use and consequently led to the use of natural alternatives where possible [1]. Phenolic and polysaccharide compounds have been successfully used as bioactive nutritional supplements due to their potent antioxidant activity and low toxicity [2,3]. In addition, these compounds have other therapeutic properties such as antibacterial, hepatoprotective, anti-inflammatory, and anticancer activities [4,5].

Seaweeds, or marine macroalgae, are increasingly attracting interest for industrial applications, as they contain multiple valuable micro- and macro-nutrients and bioactive compounds [6]. Brown seaweed is a rich source of polysaccharides and phenolic compounds with unique properties and biological activities [7,8]. These compounds are structurally unique to seaweed and are already used in a range of industries, including the pharmaceutical, medical, and food sectors [6]. The phenolics extracted from brown seaweed exhibit potent antioxidant activity [9] whereas its polysaccharides, particularly fucoidan (a sulphated polysaccharide mainly containing L-fucose as a monomer), demonstrate antioxidant activity and a range of bioactivities including anticoagulant, antithrombin, antiproliferative, anti-inflammatory, immunostimulatory, and anticancer activities against different cell lines (B-16, CT-26, HL-60, HepG2, A549, HeLa, and PC-3) [10,11]. Because of these biological effects, the phenolics and fucoidans derived from seaweed have potential applications as natural antioxidants and bioactives in food, nutritional supplements, and medicine [11].

The extraction of phenolics and polysaccharides from brown seaweed has been widely investigated. The main method for phenolic extraction is the use of organic solvents (e.g., ethanol or methanol) [12] while alkali (Na_2_CO_3_) and acid (HCl) extraction are industrially applied for extracting alginate and fucoidan, respectively [13,14,15]. Although these methods efficiently extract the target compounds, the use of organic solvents and chemicals is not green and can degrade sensitive phenolic components, making multiple-product recovery unfeasible. Other methods such as ultrasound- [16,17,18] and microwave-assisted [19,20] extraction have been proposed to improve compound yields and shorten extraction times, as they employ ultrasound energy or electromagnetic waves to break down the cellular structure of the seaweed, enhancing the release of these compounds [19]. However, the industrial application of these processes is limited due to challenges at large-scale production [21]. Supercritical fluid extraction [19,22] and subcritical water extraction [23,24] have been developed as green eco-friendly alternatives for extracting phenolics and polysaccharides. These methods efficiently extract intracellular compounds but require relatively a high pressure (30–200 bar) and temperature (150–210 °C), and are expensive [22,25].

The development of green, efficient, and cost-effective extraction methods is important for food safety, public health regulations, environmental protection, and the demand for minimizing energy consumption. Aqueous enzymatic-assisted extraction, using water as the extraction solvent, has emerged as an eco-friendly and efficient method that avoids solvents and strong acids or bases [26,27]. Cellulase, protease, and/or pectinase enzymes in tailored combinations can be used to hydrolyse the cell wall, resulting in cell disruption, facilitating the release of cell components [27]. This method is conducted at mild temperatures and pressures, lowering energy consumption and preventing the degradation of the extracted compounds [27]. Moreover, this method avoids the use of toxic chemicals while also producing high-quality products [28,29]. Enzymatic extraction has been employed for the extraction of fucoidan from *Nizamuddinia zanardinii* [19] and *Kjellmaniella crassifolia* [30], the extraction of protein from *Caulerpa prolifera* [31], the extraction of laminarin from *Ecklonia maxima* [32], agar extraction from *Gelidium sesquipedale* [33], and phenolic extraction from Danish seaweeds [34]. However, enzyme-assisted extraction has been previously used to extract one target component only (e.g., polysaccharides, proteins, or phenolics). A biorefinery approach requires the extraction of multiple components from biomass simultaneously so as to minimize waste and create maximum value from a single biomass. In order to move toward a novel biorefinery approach this study applied enzyme-assisted aqueous extraction to the simultaneous extraction of two important bioactive classes (phenolics and polysaccharides) from the seaweed *Padina gymnospora*.

*P. gymnospora* is frequently found in coastal areas worldwide. Studies have reported that *P. gymnospora* is a rich source of antioxidants [35,36], but this seaweed is underutilized and no study has optimized the conditions for extracting its bioactive compounds, particularly using a biorefinery approach where multiple bioactive classes are extracted in one step. Therefore, this study aimed to develop an efficient aqueous-only enzymatic extraction process for the simultaneous extraction of phenolics and polysaccharides from *P. gymnospora*. Different enzymes (cellulase, pectinase, and protease), individually and in combination, were screened to be used in enzyme-assisted extraction. A response surface methodology (RSM) was subsequently employed to optimize the extraction conditions (temperature, extraction time, enzyme amount, and water-to-sample ratio) for maximizing the simultaneous recovery of phenolics and polysaccharides. The extract was then fractionated to obtain different polysaccharide- and phenolic-rich fractions. Finally, the antioxidant activities were evaluated for each fraction using a 2,2-Diphenyl-1-picrylhydrazyl (DPPH) assay.

## 2. Results and Discussion

### 2.1. Effect of Different Enzymes

The current work examined the simultaneous extraction of phenolics and polysaccharides from *P. gymnospora* using enzyme-assisted extraction. To evaluate the efficiency of the enzymatic extraction method, the extraction of phenolics and polysaccharides using conventional methods was also conducted and compared with the enzymatic method. The results showed that the content of phenolics and polysaccharides obtained from the conventional extraction methods was 14.06 ± 0.47 mg GAE/g and 421.60 ± 7.60 mg/g, respectively. These values were set as a recovery of 100%, to which other extraction efficiencies were compared. The enzymatic-assisted extraction resulted in significantly higher polysaccharide and phenolic recoveries than the extraction using distilled water without enzyme did (Table 1). This is because the enzymes partially degrade seaweed cell walls, promoting the release of phenolic and polysaccharide compounds [30,37]. Among the enzymes examined, Alcalase showed the highest efficiency for the simultaneous extraction of phenolics and polysaccharides from *P. gymnospora*, with polysaccharide and phenolic recoveries of 65.93% and 74.96%, respectively; followed by a mixture of Alcalase and Pectinex (1:1, *v*/*v*), Alcalase:Cellulast (1:1, *v*/*v*), Cellulast, Cellulast:Pectinex (1:1, *v*/*v*), and Pectinex. The cellular structure of seaweed is a complex and includes different components (e.g., cellulose and protein). Therefore, this study combined different enzymes known to degrade these materials in order to test their efficacy in terms of extraction. However, the combination of different enzymes exhibited a lower polysaccharide and phenolic recovery than that obtained with Alcalase alone. This might be because the Cellulast and Pectinex are less efficient cell wall-degrading enzymes than Alcalase for *P. gymnospora*. The Alcalase-assisted extraction demonstrated a lower phenolic and polysaccharide extraction efficiency than the conventional ethanol extraction method (a phenolic and polysaccharide recovery of <100%). To further enhance its extraction efficiency, the extraction conditions were then optimized using RSM to maximize the phenolic and polysaccharide contents.

### 2.2. Optimization of Extraction Conditions

A BBD-RSM model was used to establish the relationships between the extraction factors and the recovery of phenolics and polysaccharides. As can be seen from Table 2, the central experiments (runs 25–27) show a small coefficient of variance (0.72% and 0.30% for phenolic recovery polysaccharide recovery, respectively), indicating the high precision and reproducibility of the experiments. Subsequently, the model terms were determined and the relationships between the extraction factors and the recovery of polysaccharides (*Y*_1_) and phenolics (*Y*_2_) are as follows:

(1) $$Y_{1}=84.17+1.35X_{1}+3X_{2}-1.62X_{3}-0.86X_{4}-4.12X_{1}^{2}-{5.28X}_{2}^{2}-2.41X_{3}^{2}-3.78X_{4}^{2}-4.87X_{1}X_{2}\;+1.45X_{1}X_{3}-3.2X_{1}X_{4}-0.37X_{2}X_{3}-{2.9X}_{2}X_{4}+0.66X_{3}X_{4}$$

(2) $$Y_{2}=97.68+2.49X_{1}-0.09X_{2}+4.12X_{3}+1.29X_{4}-6.04X_{1}^{2}-6.30X_{2}^{2}-{15.79X}_{3}^{2}-8.12X_{4}^{2}+2.92X_{1}X_{2}\;+{5.51X}_{1}X_{3}-0.39X_{1}X_{4}-3.70X_{2}X_{3}+1.65X_{2}X_{4}+{6.07X}_{3}X_{4}$$

**Figure 1:**
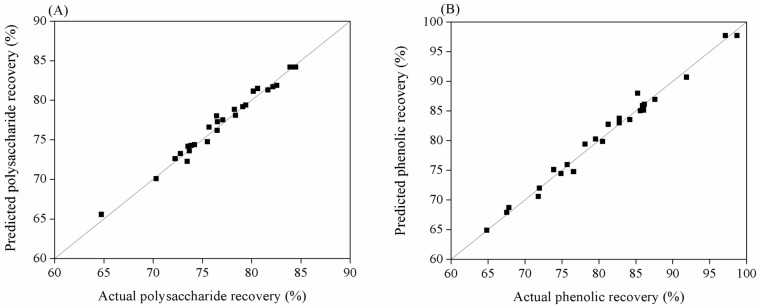
Correlation between experimental and predicted values of polysaccharide (**A**) and phenolic recovery (**B**).

An ANOVA was subsequently carried out to evaluate the statistical significance of the RSM models (Equations (1) and (2)). As shown in Table 3, low *p*-values (*p* < 0.0001) were obtained for two models, signifying the statistical significance of the established models at the 95% confidence level. Furthermore, the coefficient of determination (*R*^2^) for model 1 (polysaccharide extraction) and model 2 (phenolic extraction) was 0.98 and 0.99, respectively, indicating the preciseness of the models in their prediction and therefore that the developed models could provide reliable results (Figure 1). Table 4 shows the *t*-test for evaluating the significance of the model terms. There was a slight difference between models 1 and 2. *X*_1_, *X*_3_, *X*_4_, $X_{1}^{2}$, $X_{2}^{2}$, $X_{3}^{2}$, $X_{4}^{2}$, *X*_1_*X*_2_, *X*_1_*X*_3_, and *X*_2_*X*_4_ were crucial factors in both the polysaccharide and phenolic extractions. *X*_2_ and *X*_1_*X*_4_ were only crucial factors in the polysaccharide extraction, whereas *X*_2_*X*_3_ and *X*_3_*X*_4_ were significant factors in the phenolic extraction alone, indicating that the influence of these model terms on polysaccharide extraction differs from their influence on phenolic extraction. Consequently, the optimal extraction conditions for both polysaccharide extraction (model 1) and phenolic extraction (model 2) were determined to simultaneously maximize polysaccharide and phenolic recovery.

### 2.3. Combined Effect of Extraction Factors

**Figure 2:**
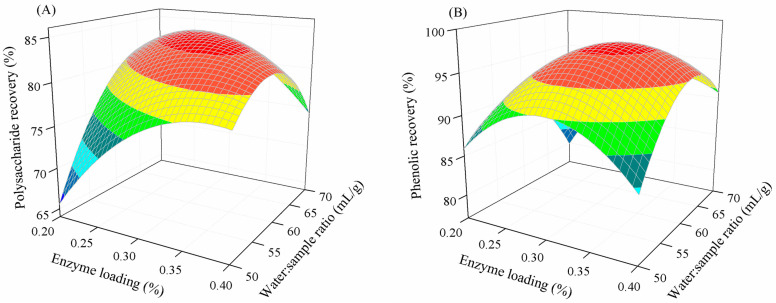
Combined effect of enzyme loading and the water-to-sample ratio on polysaccharide (**A**) and phenolic recovery (**B**).

Figure 2A and Figure 2B, respectively, illustrate the combined effect of enzyme loading and the water-to-sample ratio on polysaccharide and phenolic recovery when maintaining other extraction factors at a constant. A similar trend was found for the extraction of polysaccharide and phenolic compounds. At a specific enzyme loading, increasing the water-to-sample ratio increased the polysaccharide and phenolic recovery. However, a further increase in the water-to-sample ratio caused the reverse effect. This could be because a sufficient amount of water is required for the optimal activity of an enzyme, but enzyme stability and activity may decrease with excess water, lowering cell disruption and extraction efficiency [28,38]. This finding corresponds to that of the enzymatic extraction of phenolics from banana peel [39], and polysaccharide extraction from *Scutellaria baicalensis* root [29] and *Corbicula fluminea* [40].

**Figure 3:**
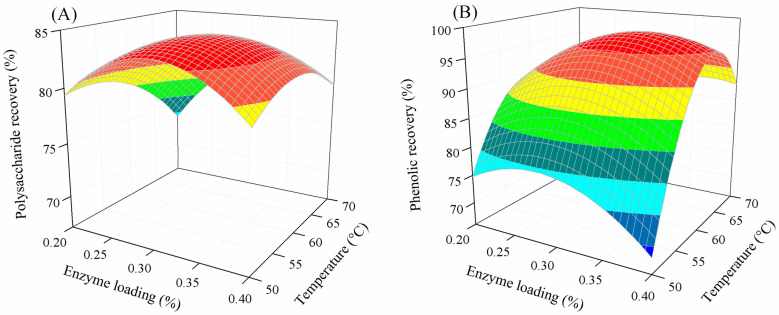
Combined effect of enzyme loading and temperature on polysaccharide (**A**) and phenolic recovery (**B**).

Figure 3 displays the combined influence of enzyme loading and temperature on polysaccharide and phenolic recovery. The influence of temperature on the polysaccharide extraction was similar to its effect on phenolic extraction. At a given enzyme loading, the polysaccharide and phenolic recovery increased with increased temperature. However, the recovery of these components decreased at elevated temperatures. This result is similar to the enzymatic extraction of polysaccharides from *Tuber aestivum* [41], *Scutellaria baicalensis* [29], *Hericium erinaceus* [42], and alfalfa [43], and phenolic extraction from banana peel [39], where the enzyme was deactivated at a high temperature. Similarly, enzyme loading had a significant influence on the extraction of polysaccharide and phenolic compounds from *P. gymnospora*. Increased enzyme loading enhanced the polysaccharide and phenolic recovery irrespective of temperature. This is because the enzyme is required for the degradation of cellular structure, facilitating the release of those components [44]. However, above a certain enzyme loading, excess enzymes reduced the extraction efficiency. This finding is similar to that of bioactive compound extraction from the brown seawweed *Sargassum muticum* [45], protein extraction from the seaweed *Gelidiella acerosa* [46], phenolic extraction from cauliflower [44] and *Trapa quadrispinosa* [47], and polysaccharide extractions from *Tuber aestivum* [41]. This lower activity might be due to enzyme aggregation at high concentrations [28].

**Figure 4:**
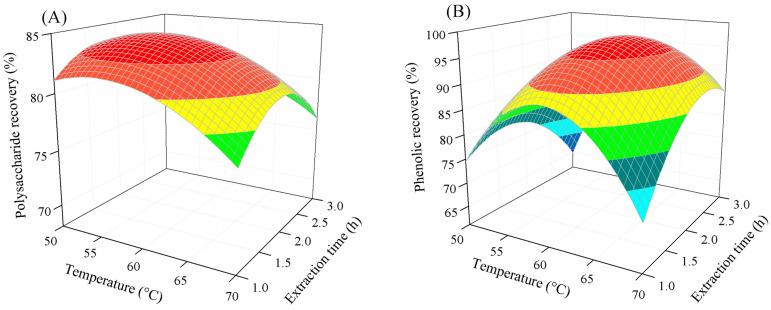
Combined influence of temperature and extraction time on polysaccharide (**A**) and phenolic recovery (**B**).

Figure 4 shows the combined influence of the extraction time and temperature on polysaccharide and phenolic recovery. The same trend was observed for the effect of the extraction time on the extraction of phenolics and polysaccharides. At a given temperature, the recovery of polysaccharides and phenolics significantly increased with an increasing extraction time, reached a peak, and then slightly reduced at longer extraction times. This could be because the enzyme requires sufficient time for extraction [28,39]. This result is in agreement with those observed for polysaccharide extraction from *Tuber aestivum* [41] and *Corbicula fluminea* [40], and phenolic extraction from the brown seaweed *Ecklonia radiata* [48] and banana peel [39].

### 2.4. Determination of Optimal Conditions for the Simultaneous Extraction of Polysaccharides and Phenolics

The RSM models (Equations (1) and (2)) were solved to determine the conditions for simultaneously maximizing polysaccharide and phenolic recovery. The optimal conditions for the simultaneous extraction were predicted to be an enzyme loading of 0.32%, water-to-sample ratio of 61.31 mL/g, temperature of 60.5 °C, and extraction time of 1.95 h, with a corresponding maximum polysaccharide and phenolic recovery of 84.46% and 97.96%, respectively. To verify the prediction, extractions were conducted under the predicted conditions, which resulted in experimental polysaccharide and phenolic recoveries of 87.45 ± 1.02% and 98.93 ± 1.49%, respectively. The predicted and experimental values were in good agreement, indicating that the established model practicability and adequately describes the relationship between the input variables and the measured response in the simultaneous extraction of polysaccharides and phenolics from *P. gymnospora*. Moreover, the large recovery of polysaccharides and phenolics obtained in the current work demonstrated the successful use of Alcalase for the simultaneous extraction of phenolics and polysaccharides from *P. gymnospora* and therefore that an enzyme-assisted extraction method is a promising approach for simultaneously extracting phenolic and polysaccharide compounds from seaweed. Brown seaweed has been widely reported as a rich source of polysaccharides with its content ranging from 25–80% depending on the species [49,50], but its phenolic content is relatively low. In this work, the polysaccharide content of *P. gymnospora* (42.16%) was comparable to other seaweed species [50,51]. The phenolic content of *P. gymnospora* (14.06 mg GAE/g) was found to be much higher than that of other seaweed species such as *Phyllospora comosa* (1.45 mg GAE/g), *Ecklonia radiata* (3.56 mg GAE/g), *Durvillaea* sp. (0.73 mg GAE/g), *Sargassum* sp. (9.56 mg GAE/g), and *Cystophora* sp. (12.83 mg GAE/g) [8]. These findings indicated that *P. gymnospora* is a promising source material for the extraction of both polysaccharides and phenolics.

### 2.5. Fractionation and Characterization

**Figure 5:**
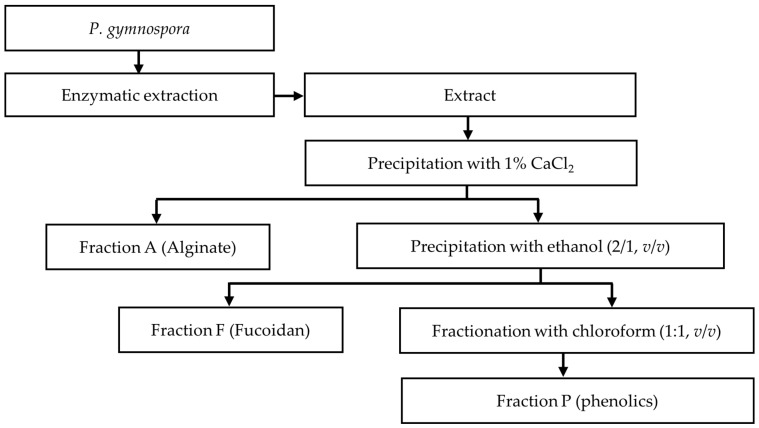
Fractionation process used to separate alginate, fucoidan, and phenolic compounds.

The extract obtained from the optimal extraction conditions was subjected to the fractionation process (Figure 5) to separate the polysaccharide and phenolic compounds. Consequently, three different fractions, namely fraction A, fraction F, and fraction P, were obtained. Fractions A (a total polysaccharide content of 82.43 ± 1.03%, based on the dry weight of sample) and F (a total polysaccharide content of 85.49 ± 0.97%, based on the dry weight of sample) were alginate-rich and fucoidan-rich fractions as they were obtained from the CaCl_2_ precipitation, followed by ethanol precipitation, respectively [52,53], while fraction P was a phenolic-rich fraction. A monosaccharide analysis was also conducted to further characterize the fractions A and F. As shown in Table 5, the monosaccharide profile of fraction A mainly comprised mannuronic acid (44.28%) and guluronic acid (26.25%), which are the main monomeric units of alginate [10], whereas fucose (54.28%) was identified as a main component in the monosaccharide profiles of fraction F. The sulphate content of faction F (17.32 ± 1.35%) was found to be higher than that of fraction A (0.21 ± 0.01%). This could be because fraction F contained fucoidan, which is a sulphated polysaccharide. This result is in agreement with previous works, since alginate and fucoidan can be separated using CaCl_2_ precipitation, followed by ethanol precipitation [52,53].

### 2.6. Anti-Radical Activity

**Figure 6:**
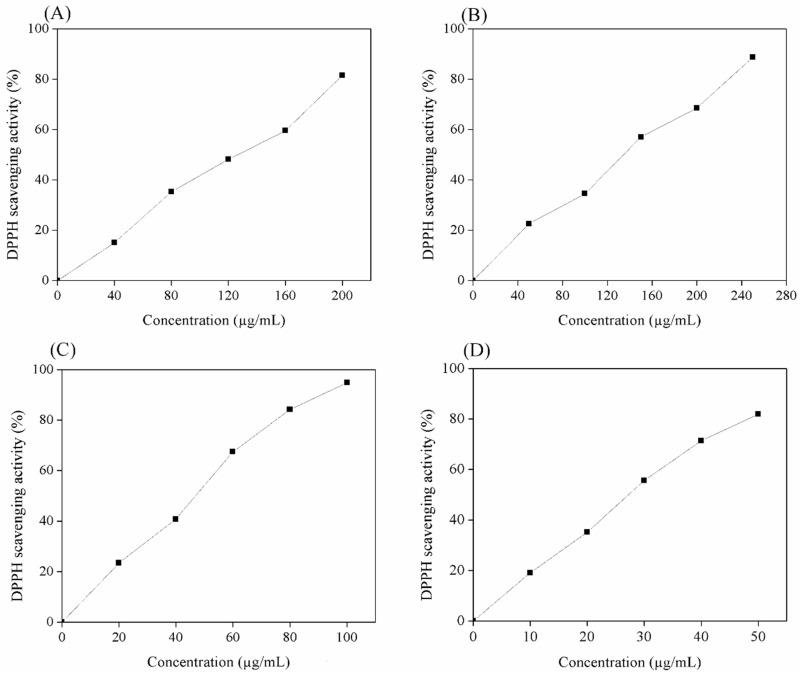
DPPH scavenging activity of fraction F (**A**), fraction A (**B**), fraction P (**C**), and ascorbic acid (**D**).

There is an increasing use of natural antioxidants in various industries, especially in food and pharmaceutical products. As a result, many studies focus on finding new antioxidant agents and/or developing efficient methods for the isolation of these compounds. This work developed an enzyme-assisted extraction method for the simultaneous extraction of phenolics and polysaccharides from a brown seaweed, *P. gymnospora*, to simplify the extraction process and reduce the use of toxic chemicals for the extraction of these compounds. The extract was then subjected to a fractionation process to obtain different fractions (alginate, fucoidan, and phenolic). The anti-radical activities of these fractions were evaluated using a DPPH scavenging activity assay. As shown in Figure 6, the extracted fucoidan, alginate, and phenolic rapidly inhibited DPPH radicals at low concentrations (µg/mL). Of the extracted samples, the phenolic fraction (fraction P) exhibited the highest DPPH scavenging activity with an IC_50_ value of 48.17 µg/mL, followed by fraction F (IC_50_ of 126.21 µg/mL) and fraction A (IC_50_ of 140.55 µg/mL) (Table 6). The higher DPPH scavenging activity of fraction F compared to fraction A could be because fraction F contained a higher level of sulphate groups, which play an important role in the biological activity of sulphated polysaccharides [54]. Notably, the phenolic and fucoidan obtained from the enzymatic extraction method showed higher DPPH scavenging activity than those extracted by conventional methods (IC_50_ values of 74.69 µg/mL and 265.68 µg/mL for the phenolic and fucoidan, respectively). This could be because the enzymatic method modified the structure of these molecules [52]. In addition, the phenolic obtained in this work exhibited a higher DPPH scavenging activity than that obtained from the red seaweed *Gelidium sesquipedale* (IC_50_ of 84.61 µg/mL), brown seaweed *Bifurcaria bifurcate* (IC_50_ of 208.5 µg/mL), and *Cystoseira humilis* (IC_50_ of 121.1 µg/mL) [55]. These findings suggest that enzymatic extraction is a promising method for obtaining highly bioactive compounds from *P. gymnospora* and that the phenolics and polysaccharides obtained from *P. gymnospora* are a potential anti-radical agent for further use.

## 3. Materials and Methods

### 3.1. Materials

Brown seaweed *Padina gymnospora* was obtained from Phu Quoc City (Kien Giang, Vietnam). The seaweed was washed with water and dried in an oven at a temperature of 40 °C for 48 h. The seaweed sample was then ground using a blender and stored at 4 °C for further experiments. Commercial enzymes, Celluclast^®^ 1.5 L (a cellulase that hydrolyses (1,4)-β-D-glycosidic linkages in cellulose and other β-D-glucans; activity of 700 endo-glucanase units (EGU)/g), Pectinex^®^ Ultra SP-L (a polygalacturonase that hydrolyzes (1,4)-α-D-galactosiduronic linkages in pectate and other galacturonans; activity of 3800 polygalacturonase units (PGNU)/mL), and Alcalase^®^ 2.5 L PF (a serine endoprotease that hydrolyses internal peptide bonds; activity of 2.5 Anson unit (AU-A)/g) were provided by Novozymes A/S (Bagsvaerd, Denmark). Ascorbic acid (99%), DPPH, ethanol, methanol, and chloroform were obtained from Sigma-Aldrich (Singapore).

### 3.2. Enzyme Screening

The simultaneous extraction of phenolics and polysaccharides from *P. gymnospora* was conducted by mixing the seaweed sample (2 g) with 0.1% (*w*/*w*, based on sample weight) of different enzymes (Cellulast, Pectinex, Alcalase, or a mixture of these enzymes), and 100 mL of distilled water at 40 °C for 2 h with stirring (400 rpm) to examine the impact of these enzymes on the extraction. Extraction using water (without enzymes) was also carried out as a control. After the completion of extraction, the extract was obtained through centrifugation at 5000 rpm for 15 min and used for the determination of phenolic and polysaccharide contents [27].

Comparative experiments using conventional extraction methods were also conducted to extract phenolic and polysaccharides from the sample. Briefly, phenolic extraction was performed by mixing the sample with aqueous ethanol (70%, *v*/*v*) with 0.1% formic acid added at a liquid: solid ratio of 20:1, a temperature of 10 °C, and extraction time of 16 h [8]. This extraction process was repeated two times to ensure maximal phenolic extraction. The ethanol extract was collected to determine the phenolic content. In order to access the total polysaccharide content, the seaweed sample was treated with 2 M trifluoroacetic acid (TFA) at 121 °C for 2 h. The resulting residues were then collected and extracted using 72% sulphuric acid (H_2_SO_4_) at room temperature for 4 h, followed by 3.2% H_2_SO_4_ at 120 °C for 4 h [56]. The polysaccharide content was subsequently determined using the phenol–H_2_SO_4_ method and fucose as a standard [56].

The recovery of phenolics and polysaccharides was then calculated as follows:

(3) $$Phenolic\;recovery\left(\%\right)=\frac{phenolic\;content\;obtained\;from\;enzymatic\;extraction\;method}{Phenolic\;content\;obtained\;from\;conventional\;extraction\;method}\times 100$$

(4) $$Polysaccharide\;recovery\left(\%\right)=\frac{Polysaccharide\;content\;obtained\;from\;the\;enzymatic\;extraction}{Polysaccharid\;content\;obtained\;from\;the\;conventional\;extraction}\times 100$$

### 3.3. Optimization of Conditions for Simultaneous Extraction

A Box–Behnken design (BBD) with 3 levels (−1, 0, 1) and 4 factors was used to investigate the effect of extraction factors (enzyme loading, *X*_1_; water-to-sample ratio, *X*_2_; extraction temperature, *X*_3_; and extraction time, *X*_4_) on the simultaneous extraction of phenolics and polysaccharides from *P. gymnospora*. The actual and coded levels of these extraction factors (input variables) are presented in Table 7. These values were selected based on the results of single-factor experiments. The extractions were performed in a sealed reactor according to the experimental design illustrated in Table 2. After the extraction was completed, the extract was collected for the determination of polysaccharide (*Y*_1_) and phenolic recovery (*Y*_2_). The relationships between the measured responses (*Y*) and input variables were then modelled using the following equation:

(5) $$Y=\beta_{0}+\sum_{i=1}^{4}\beta_{i}X_{i}+\sum_{i=1}^{4}\beta_{ii}X_{i}^{2}+\sum_{i=1}^{3}\sum_{j=2}^{4}\beta_{ij}X_{i}X_{j}\;$$

where *β*_0_, *β_i_*, *β_ij_*, and *β_ii_* are the constant, linear, interaction, and quadratic coefficients, respectively. These model terms were determined using Minitab 16 software (Minitab Inc., State College, PA, USA). The established model was then used to determine the optimal extraction conditions for simultaneously maximizing the recovery of phenolics and polysaccharides.

### 3.4. Fractionation of Extract

The extract obtained from the optimal extraction conditions was subjected to a fractionation process to obtain alginate (fraction A), fucoidan (fraction F), and phenolic (fraction P)-rich fractions, as shown in Figure 5. Briefly, 1% CaCl_2_ was added to the extract to precipitate calcium alginate. The calcium alginate was then converted to sodium alginate by dissolving it in Na_2_CO_3_, followed by precipitation with ethanol (2:1, *v*/*v*). The resulting supernatant obtained after precipitation with CaCl_2_ was mixed with ethanol (2:1, *v*/*v*) and placed at 4 °C overnight. The solution was then centrifuged to obtain the precipitate (the fucoidan-rich fraction) whereas the supernatant was collected, ethanol removed, and fractionated with chloroform (1:1, *v*/*v*) to obtain the chloroform fraction (fraction P). All fractions were subsequently freeze-dried and used for further experiments [52,53].

### 3.5. Analysis

#### 3.5.1. Determination of Phenolic Content

Total phenolic content (TPC) was estimated using the Folin–Ciocalteu method [57] with minor modifications. Briefly, 0.5 mL of the extract was mixed with 5 mL of 10% Folin–Ciocalteu reagent and incubated for 3 min. A total of 4 mL of Na_2_CO_3_ (2%) was added to the reaction mixture and incubated for 1 h in the dark. The mixture absorbance was determined at 765 nm using a spectrophotometer (V-730, Jasco, Easton, MD, USA). Gallic acid (0–100 μg/mL) was used to establish a standard curve (*y* = 0.0135*x* + 0.0131, *R*^2^ = 0.9996) to calculate the TPC. The TPC was shown as the gallic acid equivalent (GAE)/gram of dry sample.

#### 3.5.2. Monosaccharide Analysis

The monosaccharide profile of the polysaccharide fractions (fractions A and F) was determined by high-performance liquid chromatography (HPLC, Agilent, Santa Clara, CA, USA) using the method from Dai et al. [58] with slight changes. Briefly, 100 µL of the polysaccharide sample (3–4 mg/mL) was mixed with 3 mL of 4 M TFA and incubated at 110 °C for 2 h. The sample was then cooled to room temperature (RT), mixed with 200 µL of methanol, and then evaporated to dryness. Methanol (200 µL) was then added and evaporated. The process was repeated three times to remove TFA. The hydrolysed sample was dissolved in water (100 µL) and mixed with 0.6 M NaOH (100 µL). The mixture (50 µL) was then mixed with 0.5 M 1-phenyl-3-methyl-5-pyrazolone (50 µL) at 70 °C for 100 min. The reaction solution was cooled to RT and neutralized with 0.3 M HCl (50 µL). The reaction mixture was then evaporated to dryness, followed by the addition of chloroform and water (1.0 mL). The solution was mixed well, and the organic layer was removed. This process was repeated thrice. Finally, the aqueous phase was collected, filtered through a 0.45 µm syringe filter, and injected into the HPLC system equipped with a ZORBAX Eclipse XDB-C18 HPLC column for analysis. The analysis was carried out at 30 °C using a mixture of 0.1 M phosphate buffer (pH 6.7) and acetonitrile at a ratio of 83:17 (*v*/*v*, %) and a flow rate of 1 mL/min. The sample was measured for its absorbance at 245 nm.

#### 3.5.3. Determination of Sulphate Content

The sulphate content of polysaccharide fractions was determined using the BaCl_2_-gelation method [59]. Briefly, the sample was hydrolysed with 1 M HCl at 100 °C for 2 h. The hydrolysed sample (25 µL) was then reacted with 50 µL of the turbidimetric reagent (containing gelatine, BaCl_2_, and NaCl) for 15 min at RT. The reaction mixture was then measured for its absorbance at 405 nm. Na_2_SO_4_ solution (in 0.5 M HCl) was employed to establish the standard curve (*y* = 0.0012*x* − 0.0248, *R*^2^ = 0.9996) to calculate the sulphate content in the sample.

#### 3.5.4. Determination of DPPH Radical Scavenging Activity

DPPH radical scavenging activity assay was employed to evaluate the anti-radical activity of the seaweed extracts [60]. Different concentrations of seaweed samples and standard (ascorbic acid) were prepared. In total, 1 mL of each sample was mixed with 1 mL of 0.2 mM DPPH solution and incubated at RT for 30 min. The mixture was then measured for its absorbance at 517 nm using a spectrophotometer (V-730, Jasco, USA). The DPPH scavenging activity was calculated as follows:

(6) $$\mathrm{DPPH}\;\mathrm{scavenging}\;\mathrm{activity}\left(\%\right)=\left(1-\frac{A_{0}}{A_{1}}\right)\times 100$$

where *A*_0_ and *A*_1_ are the absorbances of the seaweed extract and the 0.02 mM DPPH solution without extract, respectively. The concentration required for 50% inhibition of DPPH (IC_50_) was subsequently estimated by plotting the DPPH scavenging activity versus the sample concentration.

### 3.6. Statistical Analysis

The DPPH scavenging activity and enzyme screening assay were conducted in triplicate and data are shown as mean ± standard deviation (SD). Analysis of variance (ANOVA) was conducted to analyse those data using SAS software (SAS Institute, Cary, NC, USA).

## 4. Conclusions

This study reports on a novel aqueous enzyme-assisted extraction method for simultaneously extracting phenolic and polysaccharide compounds from *P. gymnospora*. Among the enzymes and combinations tested, Alcalase alone exhibited the highest activity for the simultaneous extraction of phenolics and polysaccharides. Through RSM, the optimal extraction conditions were determined as an enzyme loading of 0.32%, water-to-sample ratio of 61.31 mL/g, temperature of 60.5 °C, and extraction time of 1.95 h, resulting in a polysaccharide and phenolic recovery of 87.45% and 98.93%, respectively, when compared with recovery from conventional, optimised, non-enzyme-assisted extraction yields using organic solvents. The extract was then fractionated further to obtain alginate-, fucoidan-, and phenolic-rich fractions. These fractions showed anti-radical activity against DPPH. Among them, the phenolic fraction demonstrated the highest DPPH scavenging activity. This study suggests that aqueous Alcalase-assisted extraction is an efficient green process for the simultaneous recovery of polysaccharides and phenolics with anti-radical activity from *P. gymnospora*. Enyzme-assisted aqueous extraction resulted in higher yields than either water only or conventional ethanol extraction methods.

## Figures and Tables

**Table 1:** Effects of different enzymes on the simultaneous recovery of phenolics and polysaccharides from *P. gymnospora*.

Enzyme	Polysaccharide Recovery (%) *	Phenolic Recovery (%) **
Cellulast	10.00 ± 0.18 ^d^	27.02 ± 1.21 ^f^
Pectinex	9.11 ± 0.22 ^f^	23.68 ± 0.85 ^g^
Alcalase	65.93 ± 0.19 ^a^	74.96 ± 2.49 ^a^
Alcalase:Pectinex (1:1)	50.93 ± 0.42 ^b^	60.67 ± 2.13 ^b^
Alcalase:Cellulast (1:1)	38.35 ± 0.38 ^c^	54.69 ± 0.78 ^c^
Cellulast:Pectinex (1:1)	9.54 ± 0.19 ^ef^	41.11 ± 0.29 ^d^
Distilled water alone	7.79 ± 0.15 ^g^	29.87 ± 0.50 ^e^

Data are shown as mean ± SD (*n* = 3). Values within a column with different letters significantly differ via an LSD test at *p* ≤ 0.05. * The polysaccharide % recovery was calculated based on the total polysaccharide content obtained from the conventional non-enzymatic extraction method (421.60 ± 7.60 mg/g). ** The phenolic % recovery was calculated based on the total phenolic content obtained from the conventional non-enzymatic ethanol extraction method (14.06 ± 0.47 mg GAE/g).

**Table 2:** Experimental design for simultaneous extraction and the obtained polysaccharides and phenolics.

Run	Variables				Responses	
	*X*_1_	*X*_2_	*X*_3_	*X*_4_	*Y*_1_ (%)	*Y*_2_ (%)
1	0	1	−1	0	80.64	73.90
2	−1	1	0	0	81.67	80.51
3	0	1	0	1	74.22	86.20
4	1	−1	0	0	76.45	85.63
5	−1	0	1	0	72.79	71.98
6	−1	0	−1	0	79.42	76.60
7	0	−1	0	−1	70.34	82.79
8	−1	0	0	−1	72.26	78.17
9	0	0	1	−1	75.70	71.83
10	1	0	−1	0	79.14	67.85
11	0	1	0	−1	82.59	79.59
12	0	−1	1	0	73.47	84.21
13	0	0	−1	−1	80.19	74.89
14	1	0	0	−1	82.19	86.06
15	0	0	−1	1	78.38	64.86
16	−1	−1	0	0	64.76	85.92
17	0	0	1	1	76.51	86.06
18	−1	0	0	1	76.56	81.29
19	1	1	0	0	73.87	91.89
20	0	−1	0	1	73.55	82.79
21	0	−1	−1	0	75.53	67.57
22	0	1	1	0	77.09	75.75
23	1	0	0	1	73.70	87.62
24	1	0	1	0	78.28	85.28
25	0	0	0	0	84.49	98.72
26	0	0	0	0	83.91	97.16
27	0	0	0	0	84.10	97.16

**Table 3:** ANOVA for the RSM models.

Source	DF	SS	MS	F Value	Probability (P) > F
Model 1 (Polysaccharide recovery)					
Model	14	553.65	39.55	49.76	<0.0001
Residual (error)	12	9.54	0.80		
Lack of fit	10	9.37	0.94	10.94	0.087
Total	26	563.19			
R^2^ = 0.98; adjusted R^2^ = 0.96					
Model 2 (Phenolic recovery)					
Model	14	2047.13	146.22	67.31	<0.0001
Residual (error)	12	26.07	2.17		
Lack of fit	10	24.45	2.44	3.01	0.275
Total	26				
R^2^ = 0.99; adjusted R^2^ = 0.97					

MS: mean square; DF: degree of freedom; SS: sum of squares.

**Table 4:** Significance of the coefficients in the RSM models.

Model Term	Model 1 (*Y*_1_, Polysaccharide Recovery)				Model 2 (*Y*_2_, Phenolic Recovery)			
	Parameter Estimate	Standard Error	*t* Value ^a^	*p* Value	Parameter Estimate	Standard Error	*t* Value ^a^	*p* Value
*β*_0_	84.17	0.51	163.53	0.000 ^b^	97.68	0.85	114.79	0.000 ^b^
*β*_1_	1.35	0.26	5.24	0.000 ^b^	2.49	0.43	5.85	0.000 ^b^
*β*_2_	3.00	0.26	11.65	0.000 ^b^	−0.09	0.43	−0.21	0.838
*β*_3_	−1.62	0.26	−6.30	0.000 ^b^	4.12	0.43	9.68	0.000 ^b^
*β*_4_	−0.86	0.26	−3.53	0.006 ^b^	1.29	0.43	0.03	0.010 ^b^
*β*_11_	−4.42	0.39	−11.45	0.000 ^b^	−6.04	0.64	−9.47	0.000 ^b^
*β*_22_	−5.28	0.39	−13.68	0.000 ^b^	−6.30	0.64	−9.87	0.000 ^b^
*β*_33_	−2.41	0.39	−6.25	0.000 ^b^	−15.79	0.64	−24.75	0.000 ^b^
*β*_44_	−3.78	0.39	−9.80	0.000 ^b^	−8.12	0.64	−12.73	0.000 ^b^
*β*_12_	−4.87	0.45	−10.34	0.000 ^b^	2.92	0.74	3.96	0.002 ^b^
*β*_13_	1.45	0.45	3.24	0.007 ^b^	5.51	0.74	7.48	0.000 ^b^
*β*_14_	−3.20	0.45	−7.18	0.000 ^b^	−0.39	0.74	−0.53	0.606
*β*_23_	−0.37	0.45	−0.84	0.420	−3.70	0.74	−5.02	0.000 ^b^
*β*_24_	−2.90	0.45	−6.50	0.000 ^b^	1.65	0.74	2.24	0.045 ^b^
*β*_34_	0.66	0.45	1.47	0.166	6.07	0.74	8.23	0.000 ^b^

^a^ t_α/2,n-p_ = t_0.025,12_ = 2.18. ^b^ *p* < 0.05 indicates the significance of the model terms.

**Table 5:** Monosaccharide composition of *P. gymnospora* extracts.

Sample	Monosaccharide Compositions (%) *					
	Mannuronic Acid	Guluronic Acid	Fucose	Glucose	Mannose	Others **
Fraction A	44.28	26.25	-	3.80	7.67	18.00
Fraction F	-	-	54.28	2.53	5.84	37.35

* Monosaccharide composition was calculated based on the total polysaccharides. ** Arabinose, galactose, xylose, and rhamnose.

**Table 6:** DPPH scavenging activity of extracted phenolics and polysaccharides.

Sample	IC_50_ Value (µg/mL)
Fraction F (enzymatic extraction)	126.21 ± 1.31 ^d^
Fraction A (enzymatic extraction)	140.55 ± 2.91 ^e^
Fraction P (enzymatic extraction)	48.17 ± 1.80 ^b^
Phenolic (ethanol extraction)	74.69 ± 1.20 ^c^
Fucoidan (acid extraction)	265.68 ± 2.02 ^f^
Ascorbic acid	28.65 ± 0.73 ^a^

Data are shown as mean ± SD (*n* = 3). Values within a column with different letters significantly differ via an LSD test at *p* ≤ 0.05.

**Table 7:** Coded and actual values of input variables in the RSM model.

Input Variables	Symbols	Levels		
		−1	0	1
Enzyme loading (%)	*X*_1_	0.2	0.3	0.4
Water-to-sample ratio (mL/g)	*X*_2_	50	60	70
Temperature (°C)	*X*_3_	50	60	70
Extraction time (h)	*X*_4_	1	2	3

## Data Availability

The data are available from the corresponding author upon request.
